# Combinations of Immunotherapy and Radiation in Cancer Therapy

**DOI:** 10.3389/fonc.2014.00325

**Published:** 2014-11-28

**Authors:** Ralph E. Vatner, Benjamin T. Cooper, Claire Vanpouille-Box, Sandra Demaria, Silvia C. Formenti

**Affiliations:** ^1^Department of Radiation Oncology, Perlmutter Cancer Center, New York University School of Medicine, New York, NY, USA; ^2^Department of Pathology, New York University School of Medicine, New York, NY, USA

**Keywords:** ionizing radiation, radiotherapy, immunotherapy, tumor immunity, clinical trials, microenvironment, abscopal effect

## Abstract

The immune system has the ability to recognize and specifically reject tumors, and tumors only become clinically apparent once they have evaded immune destruction by creating an immunosuppressive tumor microenvironment. Radiotherapy (RT) can cause immunogenic tumor cell death resulting in cross-priming of tumor-specific T-cells, acting as an *in situ* tumor vaccine; however, RT alone rarely induces effective anti-tumor immunity resulting in systemic tumor rejection. Immunotherapy can complement RT to help overcome tumor-induced immune suppression, as demonstrated in pre-clinical tumor models. Here, we provide the rationale for combinations of different immunotherapies and RT, and review the pre-clinical and emerging clinical evidence for these combinations in the treatment of cancer.

## Introduction

This review aims at providing the reader with both the rationale for and the emerging information regarding pre-clinical and clinical testing of combinations of different immunotherapies and radiotherapy (RT). We will first provide a summary of the main mechanisms cancer harnesses to evade the control of the immune system, then we will describe some of the available evidence for the effects of ionizing radiation on the immune system. We will then focus on examples of clinical studies built on this background and share some of the preliminary results that are emerging. Hopefully, this review will succeed at motivating more pre-clinical and clinical research in the novel field of combined radiation and immunity.

## Cancer’s Cross-Talk with the Host’s Immune System

The adaptive human immune system can specifically recognize up to 10^12^ unique antigens, allowing T-cells to discriminate between transformed cells and normal self (1–3). There is evidence in animal models, and indirect evidence in human beings, that a competent immune system can selectively eliminate cancer cells and protect against the development of tumors (4–9). This evidence is corroborated by the increased incidence of malignancies in immune-suppressed individuals such as AIDS patients and recipients of allograft transplants (10–13). This raises the question: if the immune system can eliminate cancers, how do cancers develop in the context of a competent immune system?

Schreiber’s modification of the immunosurveillance hypothesis addresses this question, proposing that tumors must undergo three processes before they become clinically apparent: elimination, equilibrium, and escape (14, 15). In the elimination phase, transformed cells are recognized by cognate CD8^+^ cytotoxic T-lymphocytes (CTLs) and are immediately eliminated through cytotoxic mechanisms such as Fas/Fas–ligand interactions and granzyme/perforin mediated killing. This process continues until some transformed cells evolve means to evade killing by CTLs. It is hypothesized that a phase of equilibrium forms between newly transformed cell clones and those effectively eliminated by CTLs (16). Eventually, cancer cells able to evade elimination by CTLs acquire more mutations, and develop unregulated growth, invasion, and metastases. Each of these steps is associated with active evasion of the immune system.

## Mechanisms for Immune Evasion

**Figure 1:**
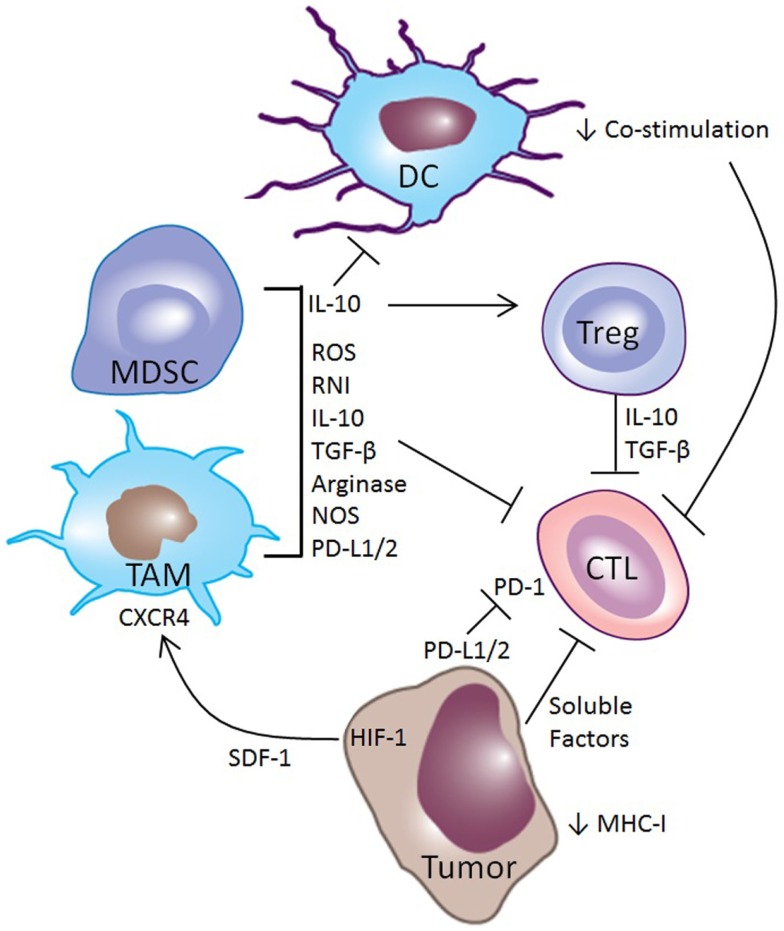
**Mechanisms of immune suppression in the tumor microenvironment**. Tumors utilize multiple mechanisms for evading the immune system. Tumor cells can down-regulate expression of MHC-I, making them poor targets for CTL mediated killing. Along with myeloid-derived suppressor cells (MDSCs) and tumor-associated macrophages (TAMs), they can express PD-L1 and PD-L2, which inhibit CTL function through the PD-1 receptor. Tumors make other soluble factors that also inhibit CTLs. Hypoxia in tumors induces HIF-1, driving the production of SDF-1, which acts as a chemokine to attract MDSCs and TAMs to the tumor microenvironment through the receptor CXCR4. These MDSCs and TAMs secrete cytokines such as IL-10 that promote a regulatory phenotype among intratumoral DCs, induce Tregs, and directly inhibit CTLs. Other myeloid-derived factors that inhibit CTL activity include TGF-β, reactive oxygen species (ROS) and reactive nitrogen intermediates (RNI), and arginase and nitric oxide synthase (NOS), which are enzymes that deplete l-arginine, an important metabolite for CTL function.

Tumors have the entire genome at their disposal for modulating and evading the anti-tumor-immune response, and their escape tends to be multi-pronged (Figure 1). One simple method of escape utilized by tumors and viruses alike, is down-regulation or inactivation of the cellular machinery responsible for MHC class I (MHC-I) antigen processing and presentation (17–20). If tumor peptide antigens are not presented by MHC-I, CTLs cannot recognize and eliminate transformed cells, although MHC down-regulation does make tumors more susceptible to NK cell cytotoxicity (21, 22).

Another common mechanism for disrupting the immune response is through interference with CTL priming, primarily through modification of the intratumoral infiltrate of dendritic cells (DCs) (3–5, 8, 9, 23). Intratumoral DCs often have an immature or regulatory phenotype that results in the presentation of tumor antigens without co-stimulation, resulting in cross-tolerance and anergy of T-cells (24–27). The importance of this mechanism in tumor-immune escape is highlighted by the close temporal correlation of antigen-specific tolerance of both CD4^+^ and CD8^+^ tumor-specific T-cells with the outgrowth of experimental tumors (6, 7, 14, 15). Additionally, regulatory DCs (regDCs) can have direct effects on tumor-immune escape, as the transfer of regDCs into tumor-bearing mice is sufficient to promote tumor growth and metastasis (16, 28).

Perhaps the most common and effective means of interfering with anti-tumor immunity is by blocking the effector function of CTLs through various mechanisms. Tumors foster the development of an immunosuppressive microenvironment by recruiting Tregs and myeloid elements – primarily tumor-associated macrophages (TAMs) and myeloid-derived suppressor cells (MDSCs) – that make TGF-β and IL-10 (29–32). These anti-inflammatory cytokines blunt anti-tumor immunity by inhibiting the cytolytic activity of CTLs. Furthermore, TAMs and MDSCs modify the metabolic milieu of the tumor microenvironment by producing arginase and nitric oxide that deplete l-arginine, an essential nutrient for T-cell function (33–35). These suppressive myeloid cells also generate reactive oxygen and nitrogen species that modify the chemokine and antigen receptors on CTLs both in the lymphoid organs and in the tumor, impairing their ability to home to tumors and kill tumor cells (36).

The tumor vasculature plays an important role in tumor-induced immune dysregulation. Tumors often outgrow their vasculature, and abnormal tumor angiogenesis results in tumor ischemia and hypoxia, which initiates recruitment of immunosuppressive myeloid cells (37). Low oxygen tension in tumors promotes an increase in hypoxia inducible factor-1 (HIF-1), which stimulates the production of stromal-derived factor-1 (SDF-1). SDF-1 acts as a chemokine, recruiting myeloid-derived cells through the chemokine receptor CXCR4 (38, 39). Furthermore, as the gatekeeper between the blood and the tumor microenvironment, the tumor vasculature plays a direct role in modulating anti-tumor immunity. Recruitment of immunosuppressive TAMs, MDSCs, regDCs, and Tregs, as well as anti-tumor CTLs, requires active engagement of the vascular endothelium in the tumor (40). While chemokine gradients attract these immune cells to the tumor, extravasation requires the expression of selectins and integrins, such as E-selectin, ICAM-1, and VCAM-1 for rolling, activation, arrest, and transmigration (41). Endothelial cells can even selectively recruit subsets of leukocytes, such as Tregs, which has been described in hepatocellular carcinoma and pancreatic cancer (42, 43). In addition to these effects, tumor cells and vascular endothelium can directly dysregulate or kill effector CTLs through engagement of the Programed Death-1 (PD-1) receptor by expressing PD-1 ligand (44–47). Current immunotherapy strategies target these mechanisms in the attempt to overcome immune escape of cancer and recover immune-rejection (48).

## Effect of Radiotherapy on Cancer Immune Response

**Figure 2:**
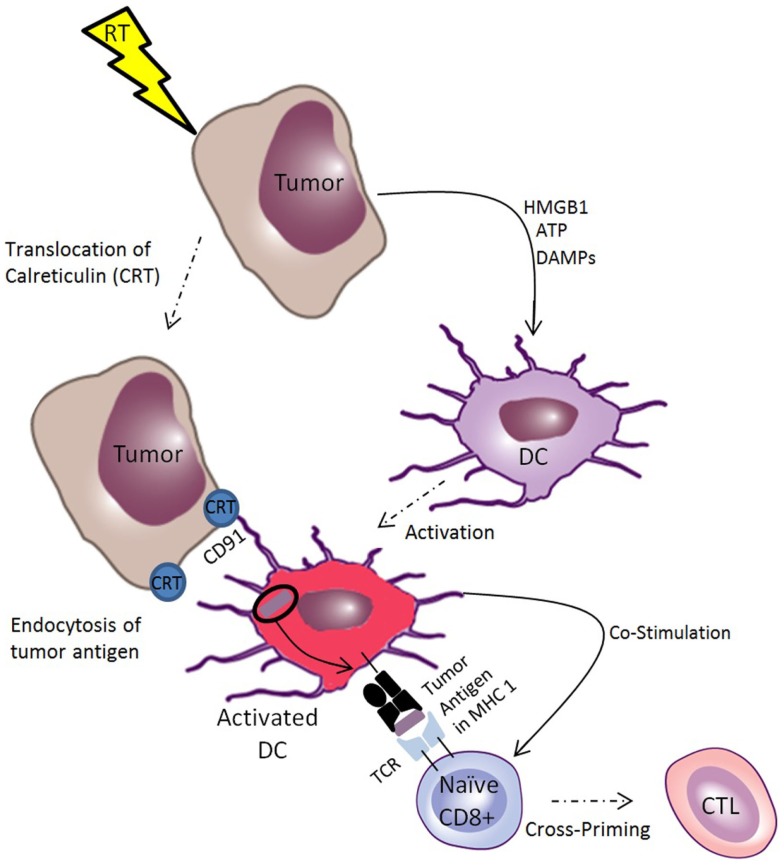
**Ionizing radiation induces immunogenic cell death of tumors, which facilitates cross-priming of CTLs**. Ionizing radiation induces translocation of calreticulin (CRT) to the tumor cell membrane, which acts as an “eat me” signal to dendritic cells (DCs), facilitating receptor mediated endocytosis through CD91. This makes tumor antigens available for cross-presentation on MHC-I for priming of tumor-specific T-cells. Radiotherapy also induces the release of danger associated molecular patterns (DAMPs), such as ATP and HMGB-1, which are endogenous immune adjuvants that stimulate DC activation, inducing DCs to provide co-stimulatory signals to naïve T-cells, facilitating cross-priming of CTLs. Together, these processes constitute immunogenic cell death of tumor cells.

Radiotherapy, while traditionally used for its direct cytocidal effect on cancer cells, also has immunomodulatory properties and can be harnessed to potentiate an immune response (49, 50) (Figure 2). Ionizing radiation causes immunogenic cell death of cancer cells, modulates antigen presentation by cancer cells, and most importantly alters the microenvironment within the irradiated field (51–54). Lymphocytes are exquisitely sensitive to ionizing radiation, and the direct effect of RT on tumor-infiltrating lymphocytes is generally cytocidal (55). This results in temporary selective ablation of immune cells within the irradiated target, depleting CTLs and NK cells directed against the tumor as well as Tregs that suppress local anti-tumor immunity. The relative importance of the effect of RT on these populations remains unclear but it is evident that the damaging effects of this physical insult are sensed by the immune system, with systemic implications.

Radiation-induced immunogenic cell death is characterized by the release of tumor antigens in the context of endogenous adjuvants that facilitates priming of anti-tumor CTLs (56). Important components of immunogenic cell death include translocation of calreticulin (CRT) to the tumor cell membrane and release of ATP and other endogenous adjuvants such as HMGB1 (57), uric acid (58), and heat-shock proteins (HSPs) (59, 60). These endogenous adjuvants act through the toll-like receptors (TLRs) to facilitate DC maturation (61–63). The role of TLRs in the mammalian immune system was first described as pattern recognition receptors that respond to pathogen associated molecular patterns (PAMPs) such as endotoxin from bacteria and double stranded RNA from viruses (64). However, there is growing evidence that the TLRs have a broader function by mediating the response to danger associated molecular patterns (DAMPs) (65). DAMPs are a larger class of molecules including PAMPs in addition to endogenous, evolutionarily conserved intracellular molecules that are released upon necrotic cell death. By linking the innate and adaptive immune system by activating antigen-presenting cells, release of DAMPs is a key aspect of immunogenic cell death mediated by RT.

Another key component of the pro-immunogenic effect of RT is the facilitation of tumor antigen uptake by DCs and cross-presentation on MHC-I (66). In fact, radiation induces MHC-I in both tumors and normal tissue (67, 68). By enhancing presentation of antigens released by its cytocidal effect, RT potentiates cross-priming of tumor-specific CTLs in the lymph nodes. Exogenous antigens can access the cross-presentation pathway by a variety of means but the most important for anti-tumor immunity is the uptake of cell-associated antigens mediated by the translocation of CRT from the endoplasmic reticulum of tumor cells to the cell surface. Ionizing radiation causes CRT to translocate to the tumor cell surface where it acts as an “eat me” signal to macrophages and DCs, which internalize CRT expressing tumor cells (69). This process is mediated by the common HSP receptor CD91, and is a necessary part of anthracycline and radiation-induced immunogenic cell death (70–72). Radiation induces the translocation of CRT on the tumor cell surface along with the release of the DAMPs HMGB1 and ATP. These signals have been shown to be necessary and sufficient in a model of radiation-induced anti-tumor immunity (73, 74).

There is evidence from both human beings and mice that tumor-associated antigens are cross-presented by DCs after RT, and this results in cross-priming of tumor-specific CTLs. By experimental necessity, much of this evidence comes from murine tumor lines transfected to express model antigens, which allow for measurement of specific CTL responses against known peptide epitopes. A single fraction of 20 Gy of ionizing radiation results in cross-presentation of an epitope from the SIY model antigen, demonstrated by an elegant set of experiments performed *in vivo* using a melanoma model (75). In a different melanoma model, both a single 15 Gy fraction of RT and fractionated RT resulted in cross-priming of CTLs detected in the tumor and tumor draining lymph nodes, with fractionated treatment resulting in a smaller degree of cross-priming (76). Other investigators have used this model to study the effect of dose and fractionation on cross-priming, and have found the number of CTLs generated correlates with the dose of radiation, but after fractionated treatment all doses of RT resulted in about the same number of primed CTLs (77). This RT induced cross-priming is dependent on TLR-4 signaling in the host (57). These findings are consistent with evidence from patients with prostate cancer who developed prostate specific CTLs after RT and vaccination with a poxviral vaccine encoding prostate specific antigen (PSA) (78).

Immunogenic cell death alone may not be sufficient to mediate a robust anti-tumor-immune response since the resident DCs within tumors maintain tolerance (3). Intratumoral injection of exogenous DCs have been used as an immune therapy for cancer, and RT has been shown to stimulate an effective anti-tumor CTL response among patients treated with this method (79–82). In some experimental systems, RT overcame the suppressive effect of tumor resident DCs by recruiting new myeloid-derived DCs that have not been exposed to the regulatory effects of the tumor microenvironment. Tumor irradiation recruits these monocyte derived DCs (mDCs) to tumors after treatment with a single large fraction of 25 Gy (83). In summary, RT induces multiple intracellular adhesion molecules (ICAMs), chemokines, and cytokines that mediate naïve DC recruitment and may at least in part subvert the immune-tolerant microenvironment characteristic of established tumors (84–86).

Furthermore, RT facilitates the recruitment of effector T-cells to tumors through the induction of chemokines. Chemokines are known to be important for the recruitment of leukocytes to tumors as part of anti-tumor immunity (87, 88). However, tumors with their immunosuppressive milieu tend to produce chemokines that recruit Tregs and other suppressive elements (89, 90). Without effective chemotaxis, lymphocytes primed against tumor antigens cannot home to tumors and carry out their effector function. CXCL16 is a chemokine that has been identified as a prognostic factor that correlates with improved survival and increased numbers of tumor-infiltrating lymphocytes in colorectal cancer and renal cell carcinoma (91–93). RT induces CXCL16 production in the 4T1 mouse breast cancer model, which mediates T-cell recruitment to tumors through the CXCR6 receptor on T-cells (94). Radiation also has effects on the tumor vascular endothelium, inducing cell adhesion molecules that further promote recruitment of anti-tumor CTLs (95). Although it does not explain the systemic immune effects of RT, chemotaxis induced by RT may partially account for the direct effects of RT on tumor control.

## The Abscopal Effect of Radiotherapy

The effects of ionizing radiation on the anti-tumor-immune response support the hypothesis that the immune system is responsible for the abscopal effect of RT. Originally described by Mole, the abscopal (from the Latin *ab* and the Greek *scopus*, away from the target) effect of radiation therapy is a phenomenon by which a primary tumor is irradiated and a response is seen at distant metastatic sites outside of the path of the radiation (96–102). Our group has generated pre-clinical evidence that it is mediated by the immune system (103–105). Even the “in field” effects of radiation have been shown to be dependent on the immune system, as CD8^+^ T cells and type I interferon are required for tumor regression after radiation therapy, since their depletion abrogates tumor control after RT (75, 83, 106–108).

Despite the observation that radiation induces effects sensed by the immune system and modulates the immune response to tumors, abscopal responses are rarely seen in clinical practice. Although there is evidence that radiation therapy alone is sufficient to provide the necessary signals for cross-priming of CTLs against tumor antigens, this adjuvant effect of radiation appears to be relatively weak. However, the rare radiation-induced systemic abscopal response can be facilitated when additional immune manipulation is added. RT primes new anti-tumor CTLs but these CTLs are usually unable to overcome the suppressive effect of the tumor microenvironment at distant untreated metastatic sites. This is the rationale for combining systemic immunotherapies with RT.

## Anti-Immunogenic Effects of RT

It must be noted that RT has anti-immunogenic effects in addition to the pro-immunogenic effects described above. There are reports that RT can impair DC function, including cross-priming (109, 110). Additionally, RT can contribute to the immune-suppressive tumor microenvironment by recruiting MDSCs and TAMs (76, 111–113). Tumor-infiltrating Tregs are also enriched after RT (77, 114). The relative importance of these immunosuppressive effects of RT remains unclear and it is likely to be model-dependent, since there are contrasting reports of RT resulting in a shift toward a macrophage mediated pro-immunogenic microenvironment (115).

## Combinations of Radiotherapy and Immunotherapy in the Clinic

There have been a number of efforts recently to combine immunotherapy with RT to augment the anti-tumor-immune effects of RT. Abscopal responses to RT alone are extremely rare, suggesting that combinations with immunotherapy may be required to sustain the pro-immunogenic effects of radiation. Similarly, only a small proportion of cancer patients derive objective benefit from currently available immunotherapies. One strategy to increase both the likelihood and duration of systemic anti-tumor immunity in response to immunotherapy is to add RT as an adjunct to bolster the immune response. When combined with RT, immunotherapeutic approaches can be broadly separated into (1) the promotion of cross-priming of tumor-specific CTLs, (2) the stimulation of immune effector function of CTLs primed by RT, and (3) neutralization of the immunosuppressive effects of the tumor microenvironment. Essentially, all current clinical approaches fall into the first two categories, with the third category primarily in the pre-clinical stage.

## Promotion of Cross-Priming of Tumor-Specific CTLs

### Growth factors to facilitate recruitment of DCs

The use of growth factors to recruit DCs from the bone marrow to the irradiated tumor was based on the very first animal model of the abscopal effect. In this model, syngeneic breast cancer cells were implanted subcutaneously into the bilateral flanks of Balb/c mice. Once the tumors grew into palpable nodules the tumor on one side was treated with RT and systemic fms-like tyrosine kinase-3 (flt-3) ligand was given concomitantly to recruit DCs from the bone marrow. The combination of RT and flt-3 ligand inhibited growth of both the irradiated tumor and the contralateral untreated tumor. This abscopal effect in a tumor nodule outside of the radiation field was demonstrated to be tumor-specific and was not observed when the experiment was repeated in nude mice, which lack T-cells, suggesting an immune-mediated mechanism (104). Due to the lack of clinical availability of flt-3 ligand, GM-CSF – another DC growth factor – was substituted when these pre-clinical studies were translated into a proof of principle pilot study at our institution for patients with metastatic solid tumors. GM-CSF increases the percentage of DCs and promotes their maturation; facilitating cross-presentation of newly released antigens after cancer cell death is achieved within the irradiated tumor. In this study, one measureable metastatic lesion was treated to a dose of 35 Gy in 10 fractions, and starting on day seven (after 1 week of radiation) GM-CSF (125 μg/m^2^) was administered subcutaneously every day for 14 days. Abscopal response was defined as a measurable response in any of the measurable lesions outside the radiation field, assessed by PET-CT. Results of this trial were reported at the American Society for Therapeutic Radiation Oncology (ASTRO) annual meeting in 2012, and a manuscript describing the long-term outcome of the treated patients is in preparation. A weakness of this study was the lack of immune-monitoring available for these patients.

### Intratumoral injection of autologous DCs

A more direct, albeit labor intensive, method for delivering DCs to the site of tumor antigen release after RT is by direct injection. For this therapy, autologous DCs are generated from mononuclear cells isolated by leukapheresis from peripheral blood by culturing these *in vitro* in the presence of cytokines and growth factors (GM-CSF). These DCs are then reintroduced directly into the irradiated tumor by injection. In one study utilizing this method, five HLA-A2^+^ patients with high-risk prostate cancer were treated with androgen suppression, 45 Gy of external beam RT and intraprostatic DC injections after fractions 5, 15, and 25. Serial prostate biopsies before and during treatment showed apoptosis of tumor cells and an increase in tumor-infiltrating CD8^+^ T-cells, as well as an increase in prostate specific CD8^+^ T-cells in the peripheral blood (116). This approach has also been used neoadjuvantly in patients with high-risk soft tissue sarcoma. Seventeen patients were treated to 50.4 Gy in 28 fractions with intratumoral injection of 10^7^ DCs, three times during treatment and once near surgery to assess for cell migration. Nine patients (53%) developed tumor-specific immune responses, which lasted up to 42 weeks with 12 of 17 patients (71%) free of progression at 1 year (117). There is one small completed randomized trial using this approach, investigating radiation therapy with and without intratumoral DC injection. Preliminary results reported 5/14 patients exhibiting an enhanced T-lymphocyte response in the experimental arm versus 2/6 in the control arm (ClinicalTrials.gov identifier: NCT01347034).

Intratumoral injection of DCs has also been used in patients with refractory hepatoma in combination with 8 Gy, single-fraction RT. All 14 patients in this study tolerated the treatment, while half of the patients had a minor or partial response clinically, and 8 patients developed an AFP specific immune response (118). There is an ongoing proof of principle trial studying the combination of RT with intratumoral DC injection in patients with malignant melanoma (ClinicalTrials.gov identifier: NCT01973322). Another recently completed phase I/II study examined the combination of intratumoral DC injection with gemcitabine and hypofractionated stereotactic body radiation therapy (SBRT) in the setting of unresectable pancreatic cancer, but results are pending (ClinicalTrials.gov identifier: NCT00547144).

### DC activation using TLR agonists

Another approach to improving T-cell cross-priming in response to RT is to activate intratumoral DCs using TLR agonists, thus improving the ability of DCs to present tumor antigens released by RT and to provide co-stimulation to naïve T-cells. This results in more robust priming and effector function of tumor-specific CTLs. Many different TLR ligands, both natural and synthetic, have been utilized in conjunction with RT to boost anti-tumor immunity. PSK, a protein-bound polysaccharide derived from the fungus *Basidiomycete coriolus versicolor* has been shown to activate NK cells and DCs through TLR2, leading to its use in conjunction with chemoRT in locally advanced rectal cancer (119, 120). Thirty patients were treated with the oral antimetabolite radiosensitizing chemotherapy S-1 in combination with neo-adjuvant radiation (20 Gy in 10 fractions) followed by radical surgery with intra-operative electron therapy (15 Gy). Patients were randomized between PSK given three times a day at a dose of 3 g/day or placebo during the neo-adjuvant external beam portion of the treatment. There was a significant increase in the percentage of NK cells in the peripheral blood and an increase in number of CTLs in the rectal mucosa, as well as a decrease in the immunosuppressive acidic protein level in the serum of patients treated with PSK (121). A suspension of heat killed *Mycobacterium obuense*, called IMM–101, also contains TLR2 agonists, which has been shown to be safe and well tolerated in human beings (122). The combination of IMM–101 and single-fraction linear accelerator based stereotactic radiosurgery is currently being tested in a single arm, phase II study in patients with previously treated metastatic colorectal cancer (ClinicalTrials.gov identifier: NCT01539824). Similarly, a hot water extract from bacillus tuberculosis called Z-100, containing polysaccharides such as arabinomannan and mannin, has immunomodulatory properties (123). This was tested in a Japanese phase III, randomized trial in patients with stage IIB – IVA cervical cancer in conjunction with standard of care chemoRT with cisplatinum. A total of 249 patients were randomized to biweekly subcutaneous injections of Z-100 or placebo and concurrent RT. Z-100 demonstrated a trend toward increased overall survival (*p* = 0.07), although the statistical power of this study was less than anticipated because survival rates were higher than expected for both arms (124). There is also an ongoing trial of the TLR4 agonist glucopyranosyl lipid A in combination with five to six fractions of RT in patients with metastatic sarcoma, (ClinicalTrials.gov identifier: NCT02180698).

TLR3 is the receptor for poly-ICLC, a synthetic double stranded RNA shown to increase the antibody response to antigen and augment the activation of NK cells, macrophages, and T-cells (125, 126). The North American Brain Tumor Consortium conducted a single-arm phase II trial of patients with recurrent anaplastic glioma, testing 20 mcg/kg poly-ICLC administered three times weekly by intramuscular injection in combination with 200 cGy daily RT to the recurrent brain tumor to a total dose of 60 Gy followed by poly-ICLC for up to 1 year, or until tumor progression. Thirty eligible patients demonstrated a 1-year overall survival of 69%, which compares favorably to the group treated with RT alone (127).

TLR9 agonists have also been the target of investigation of combined immunoradiotherapy. Brody et al. injected the CpG DNA PF-3512676 into the tumors of 15 patients with low-grade B-cell lymphoma treated concurrently with low-dose RT, resulting in a 27% response rate (128). The success of this approach led to its application in mycosis fungoides in a phase I/II study that demonstrated a 33% response rate and a trend toward a reduction of CD25^+^ T-cells (primarily Tregs) and dermal DCs in the clinical responders (129).

Imiquimod is a synthetic imidazoquinoline, which specifically activates TLR7, expressed by both plasmacytoid DCs and CD11c^+^ myeloid-derived DCs (130). In pre-clinical models, we have shown that RT in combination with imiquimod significantly improves survival of tumor-bearing mice treated with either modality alone, and based on these results we initiated an ongoing phase I/II study of imiquimod and RT for patients with breast cancer metastatic to the skin or recurrent on the chest wall (ClinicalTrials.gov identifier: NCT01421017) (131, 132). Imiquimod is also being used in a pilot study in combination with concurrent radiation in an attempt to improve outcomes in diffuse intrinsic pontine glioma, a pediatric brain tumor with a poor prognosis (ClinicalTrials.gov identifier: NCT01400672).

### Cytotoxic gene therapy

Cytotoxic gene therapy delivered *in situ* is a different tactic for improving the radiation-induced anti-tumor-immune response. This method employs intratumoral injection of recombinant viruses carrying genes that induce tumor-specific cell death, which complements the immunogenic cell death induced by RT. Cancer gene therapy using herpes simplex virus thymidine kinase (HSV-tk) in combination with gancyclovir, acyclovir, or valacyclovir to induce tumor cell death and anti-tumor immunity in combination with RT has been used with moderate success in patients with prostate cancer. After completing a phase I trial to establish safety in 18 men, this approach was tested in 33 men with intermediate and high-risk features in combination with definitive RT and anti-hormonal therapy (133). With a median follow-up of 26 months, mean percentages of DR^+^CD8^+^ T cells were increased at all time-points up to 8 months with DR^+^CD4^+^ T cells increased later and sustained longer until 12 months (134). The same group is conducting three parallel trials as salvage treatment in patients who progress after RT, as neo-adjuvant treatment prior to radical prostatectomy, and in combination with definitive RT. The addition of RT significantly increased both CD4^+^ and CD8^+^ T-cells in peripheral blood when compared to the methods lacking combined RT, adding support to combined modality therapy (135). This led to the initiation of a phase III multi-center randomized trial that will be very important in establishing the efficacy of this approach (ClinicalTrials.gov identifier: NCT01436968). We will also learn of the potential activity of this approach in patients with malignant glioma (ClinicalTrials.gov identifier: NCT00589875, NCT00751270) and pediatric brain tumors (ClinicalTrials.gov identifier: NCT00634231), and using a similar approach in pancreatic cancer (ClinicalTrials.gov identifier: NCT00638612), with completion and reporting of ongoing trials.

### Vaccines

Therapeutic cancer vaccines promote anti-tumor immunity by stimulating T-cell priming against tumor antigens, or peptide antigens thought to be specific or cross-reactive with tumors. This is another method that acts in parallel with RT for inducing anti-tumor immunity, and is often given with exogenous immunostimulatory adjuvants that promote cross-priming of T-cells against the vaccine antigen as well as antigens released by RT. Pre-clinical studies support the synergistic effect of therapeutic vaccination with RT. For example, a combination of 8 Gy delivered with a recombinant vaccinia-carcinoembryonic antigen vaccine (CEA) resulted in rejection of CEA expressing colon cancer, an effect that was not observed when the treatments were given individually (136). Human studies mimic these results.

One powerful effect of tumor vaccines is the ability to jumpstart the anti-tumor-immune response to both vaccination and RT, inducing a phenomenon known as an “antigen cascade” or “epitope spreading” (137). Initially discovered in models of autoimmune disease, and more recently described after administration of peptide-based cancer vaccines, epitope spreading describes the generation of T-cells specific for distinct and non-cross reactive tumor antigens after vaccination against known antigens (138). This phenomenon was particularly well characterized after peptide-based vaccination for prostate cancer that was administered concurrently with standard definitive RT. In this phase II trial, 30 men with clinically localized prostate cancer were randomized 2:1 to receive vaccine plus prostate directed RT or RT alone. The vaccine consisted of a recombinant vaccinia viral vector coding for PSA and the co-stimulatory molecule B7.1, and was administered concurrently by subcutaneous injection with GM-CSF and low-dose IL-2, followed by monthly booster vaccination with recombinant fowlpox-PSA. Eight patients had extensive analysis of their PBMCs for tumor-specific T-cell responses, and six of these eight patients developed T-cells specific for multiple tumor-associated antigens that were not included in the vaccine, such as PAP, MUC-1, PSMA, and PSCA (78). This suggests vaccination against a single tumor antigen along with RT can spark an antigenic cascade that results in an immune response against many endogenous tumor antigens. Most vaccine trials do not specifically incorporate RT for its immunogenic properties, and will not be described here.

## Stimulation of Immune Effector Function of CTLs

### Cytokines to bolster immune effector function

One approach to improving the efficacy of tumor-specific T-cells induced by RT is to bolster the effector function of these T-cells and other leukocytes through the use of cytokines. Interferons are a group of proteins that are secreted by DCs, lymphocytes, macrophages, fibroblasts, and other leukocytes, that increase the activity of immune effector cells and make cancer cells into better immune targets by increasing antigen processing and presentation (139). The combination of interferon alpha and chemoradiation provides a survival advantage over chemoradiation alone in early studies of patients with completely resected pancreatic cancer (140). Unfortunately, the treatment is toxic, with 95% of patients developing grade 3 or higher toxicity. This has led to the premature closure of ACOSOG Z05031, a randomized trial assessing a similar treatment strategy, and until now, other randomized trials have failed to show a benefit to combined adjuvant chemoradiation with immunotherapy for resected pancreatic cancer (141, 142).

Similar toxicity was observed when tumor necrosis factor-alpha (TNF-α) in combination with radiation was tested for locally advanced and metastatic tumors. This phase I trial resulted in a 23% patient withdrawal rate due to major toxicity (143). In an attempt to improve the tolerability of TNF-α therapy, TNFerade was developed; a replication deficient adenovector that expresses human TNF-α under the control of the radiation-inducible Egr-1 promoter. This was first tested in human beings in conjunction with radiation in a phase I trial involving 36 patients with solid tumors, of whom 70% had an objective response with no dose-limiting toxicities (144). Phase I and II studies were subsequently conducted in soft tissue sarcoma, rectal cancer, pancreatic cancer, esophageal cancer, and recurrent head and neck cancer (145–149). The promising results in the locally advanced pancreatic cancer setting led to a multi-institutional, phase III randomized trial of concurrent fluorouracil and RT with or without TNFerade. Three hundred and four patients were randomized 2:1 in favor of TNFerade treatment. Lack of benefit in progression-free or overall survival dampened the optimism for this therapeutic approach in this tumor setting (150).

Interleukin 2 (IL-2) is a cytokine that is necessary for the growth, proliferation, and differentiation of T-cells to become antigen-specific CD4^+^ and CD8^+^ T-cells. IL-2 has been used with meager success for both melanoma and renal cell carcinoma (151, 152). Pre-clinical studies demonstrated increased cytokine release (153, 154) and up-regulated expression of MHC-I (68), B7.1 (155), and Fas/CD95 (156, 157) with the addition of radiation. This inspired a phase I study combining IL-2 and SBRT for patients with metastatic renal cell carcinoma and melanoma in which 2/3 of the patients demonstrated a response, and immune-monitoring looking at cryopreserved PMBCs showed a significantly greater frequency of proliferating CD4^+^ T cells with an early activated effector memory phenotype (158).

A phase II study is ongoing, looking at the combination of IL-2 and SBRT in patients with metastatic renal cell carcinoma to assess for both a local and systemic response with the rationale that large fractions of radiation (8–20 Gy) in combination with IL-2 will increase antigen presentation and immune stimulation (ClinicalTrials.gov identifier: NCT01896271). A similar strategy is being employed by the Dutch in the setting of oligometastases in an ongoing phase I trial (ClinicalTrials.gov identifier: NCT02086721). In an attempt to decrease the toxicity of IL-2 treatment, there is an industry sponsored phase II trial combining SBRT with MSB0010445, a modified IL-2 cytokine bound to a monoclonal antibody specific for DNA, which localizes the treatment to necrotic cells (ClinicalTrials.gov identifier: NCT01973608).

### Enhancement of T-cell co-stimulation

Co-stimulation refers to the activating signals delivered to T-cells – along with antigen-specific stimulation through engagement of the T-cell receptor – that are required for effective priming and anti-tumor effector function (159). More generally, this is an important tool used by the immune system to prevent autoimmunity by ensuring the presence of DAMPs at the time of T-cell priming. The use of TLR agonists, described above, indirectly enhances co-stimulation and priming of tumor-specific T-cells; however, agonists of the co-stimulatory receptors can be utilized to directly promote co-stimulation and improved activation and effector function of anti-tumor T-cells. There are two general families of co-stimulatory molecules, the B7/CD28 immunoglobulin family and the TNF/TNFR family (160). The stimulatory B7-family members include CD80 (B7-1) and CD86 (B7-2), which stimulate T-cells through CD28, and CD275 (ICOS-L), which acts through CD278 (ICOS) (161). The TNF/TNFR family includes CD154 (CD40L), CD252 (OX40L), CD70, and CD137L (4-1BBL), which signal through CD40L, CD134 (OX40), CD27, and CD137 (4-1BB), respectively.

Many of these co-stimulatory molecules and pathways are already targets for anti-cancer therapy, but there is more limited experience combining them with RT. Monoclonal antibody agonists of CD40 improve the efficacy of DC based immunotherapy (162), and are showing promise in combination with standard chemotherapy (163–165). Antibody agonists to 4-1BB are also showing promise as immunotherapy, especially in combination with other immunotherapies (166, 167). For example, overall survival was improved in a murine glioma model when radiation was combined with a 4-1BB agonist and blockade of cytotoxic T-lymphocyte antigen-4 (CTLA-4). As predicted, treatment with the triple therapy resulted in a higher density of CD4^+^ and CD8^+^ tumor-infiltrating lymphocytes when compared to RT or either immunotherapeutic agent alone (168). Furthermore, 4-1BB activation augments the effects of RT in the murine M109 lung cancer and EMT6 breast cancer models, in which a single dose up to 15 Gy or fractionated RT up to 20 Gy slowed tumor growth to a significantly greater extent in combination with an antagonist antibody to 4-1BB (169).

OX40 is one of the more powerful co-stimulatory receptors expressed on activated T-cells, and signaling through OX40 is capable of breaking tolerance (170). Signaling through OX40 by OX40 ligand or monoclonal antibody agonists stimulates T-cells to proliferate, produce cytokines, and improve their effector function (171, 172). In a pre-clinical model of lung cancer transfected with an experimental antigen, a combination of a monoclonal antibody OX40 agonist with a single fraction of 20 Gy resulted in improved tumor response and increased antigen-specific CD8^+^ T-cells that were not observed with either treatment alone (173). There is an ongoing phase Ib clinical trial testing the effect of cyclophosphamide, RT, and an antibody agonist of OX40 in patients with metastatic prostate cancer (ClinicalTrials.gov identifier: NCT01303705). In a way, this is actually two types of immunotherapy combined with RT. Although cyclophosphamide is a conventional chemotherapy, when given in low doses it tends to selectively deplete Tregs over effector T-cells, thus removing a barrier from the anti-tumor-immune response (174, 175). This effect of cyclophosphamide was first discovered 40 years ago, and is only now being utilized in clinical trials to modulate anti-tumor immunity (176). Cyclophosphamide (300 mg/m^2^) is administered intravenously on day 1, followed by a single 8 Gy dose of RT on day 4 treating up to three osseous metastases along with the OX40 agonist treatment, which is repeated every 2 days for a total of three doses. There is a similar study of patients with metastatic breast cancer combining OX40 agonist treatment with SBRT utilizing doses ranging from a single fraction of 15 Gy up to two fractions of 20 Gy (ClinicalTrials.gov identifier: NCT01642290). Safety and immune correlates are the primary outcome measures of these trials, but early results have not yet been reported.

### Checkpoint blockade to bolster CTL effector function

A reciprocal approach that is also effective for boosting effector T-cell function is to block the immune checkpoints that counteract endogenous co-stimulation of activated T-cells (177). Immune checkpoints are a collection of endogenous mechanisms for preventing unchecked T-cell activation and runaway immune responses after effector T-cells have neutralized an infectious or neoplastic threat. Checkpoint receptors, including CTLA-4 and PD-1, are up-regulated on activated T-cells and transmit inhibitory signals, which suppress T-cell proliferation and function (159). For example, in addition to the co-stimulatory receptor CD28, activated T-cells also express CTLA-4, which directly competes for binding to the co-stimulatory ligands CD80 and CD86 (178). CTLA-4 acts as a natural checkpoint to prevent indefinite activation of T-cells, and inhibition of this immune checkpoint with a monoclonal antibody antagonist to CTLA-4 shifts the balance of co-stimulation toward increased proliferation and function of activated T-cells, including tumor-specific CTLs.

There is extensive data, both pre-clinical and from patients, demonstrating the effectiveness of CTLA-4 blockade. Monotherapy with the CTLA-4 antagonist ipilimumab resulted in a significant increase in overall survival of patients with metastatic melanoma in two large randomized trials, and is now one of the most promising immunotherapeutic agents (179, 180). In our pre-clinical studies, CTLA-4 blockade acts synergistically with RT to induce an abscopal response to RT in murine models of poorly immunogenic breast cancer and colon cancer (105). Importantly, these studies demonstrated that oligofractionation of RT (8 Gy × 3) was more effective at inducing an abscopal response than a single large fraction of 20 Gy or more fractionated treatment (6 Gy × 5). We are currently testing this approach in an ongoing phase I/II clinical trial for patients with metastatic non-small cell lung cancer (ClinicalTrials.gov identifier: NCT02221739) and in a phase III, randomized trial for patients with metastatic melanoma (ClinicalTrials.gov identifier: NCT01689974). In the lung cancer study, patients with at least two measurable sites of disease are treated with 30 Gy in five consecutive fractions to one metastatic site with concurrent ipilimumab (3 mg/kg) administered intravenously every three weeks for four cycles starting within 24 h of the first fraction of RT. The same treatment is administered in the melanoma study but half of the patients are randomized to treatment with ipilimumab alone. The primary endpoints are the safety of the combined therapy and presence of an abscopal response in measurable metastatic sites on follow-up PET/CT, determined by immune-related response criteria using the modified WHO criteria.

Clinical trials using this same combination, but with a different treatment schedule and RT regimen have been recently published. An open-label phase I/II trial for men with metastatic castration-resistant prostate cancer tested escalated doses of ipilimumab from 3 mg/kg up to 10 mg/kg in 33 patients with or without a single 8 Gy dose directed at one to three osseous metastases. The highest dose of ipilimumab was well tolerated and an additional 34 patients were treated with concurrent radiation with only 25% of patients demonstrating progressive disease (181). To further test this treatment approach, a double-blind, randomized multi-center trial was conducted including 799 men with castration-resistant prostate cancer who progressed on docetaxel (182). Patients were treated with a single fraction of 8 Gy to one to five sites of osseous metastases and randomized to subsequent treatment with either 10 mg/kg of ipilimumab or placebo within 2 days of RT and continued every 3 weeks for up to four doses. The regimen was well tolerated but there was no difference in overall survival in the population as a whole. However, in subset analysis there was an improvement in overall survival of patients with a smaller burden of metastatic disease, demonstrated by alkaline phosphatase less than 1.5 the upper limit of normal, hemoglobin greater than 11 g/dL, and an absence of visceral metastases. While only limited clinical trial data are available in the published literature justifying a combined approach, this is an area of extremely active research (Table 1).

**Table 1:** **Active clinical trials testing the combination of ipilimumab and radiotherapy**.

Clinicaltrials.gov identifier	Disease site	Design	Phase	Primary outcome measure	Radiation dose/timing	Institution(s)
NCT01557114	Melanoma (stage III/IV)	1 arm: ipi with RT	I	Maximum tolerated dose	9, 15, 18, 24 Gy in three fractions with concurrent ipi	Gustave Roussy
NCT01996202	Melanoma (locally advanced or unresectable)	Two cohorts: (A) resected high-risk patients or (B) neoadjuvant, locally advanced	I	Safety and tolerability	No data provided	Duke University
NCT01565837	Melanoma (oligometastatic but unresectable)	1 arm: ipi with SRT	II	Safety and tolerability	SRT one to five lesions with third cycle of ipi	Comprehensive cancer centers of Nevada
NCT01703507	Melanoma (brain metastases)	Two arms: (A) ipi with WBRT or (B) ipi with SRS	I	Maximum tolerated dose	(A) WBRT weeks 1 and 2 (B) SRT week 1. Ipi delivered weeks 1, 4, 7, 10	Thomas Jefferson University
NCT01449279	Melanoma (stage IV)	One arm: ipi with RT	I	Safety	Pallitive RT within 2 days of ipi	Stanford
NCT01689974	Melanoma (stage IV)	Two arms, randomized: ipil ± RT	II	Tumor response	6 Gy × 5 given on consecutive treatment days starting on day 1 with Ipi on day 4	New York University
NCT01497808	Melanoma (metastatic)	One arm: ipi with SRT	I/II	Dose-limiting toxicity	SRT 1 lesion prior to ipi	University of Pennsylvania
NCT01970527	Melanoma (stage IV)	One arm: SRT before ipi	II	Immune-related response, toxicity and survival	3 fractions of SRT between days 1–13 followed by ipi	University of Washington/NCI
NCT01935921	Head and neck (stage III–IVB)	One arm: ipi, cetuximab and RT	I	Safety and tolerability	IMRT 5 days a week for 7 weeks with cetuximab and ipi at week 4 for 3, 21 day courses	NCI
NCT01711515	Cervical cancer (stage IB–IVA)	One arm: ipi, cisplatinum and RT	I	Safety and tolerability	Standard of care chemoradiation followed by 4, 21 day cycles of ipi within 2 weeks	NCI
NCT02107755	Melanoma (metastatic)	One arm: ipi followed by SRT	II	Progression-free survival	Ipi weeks 1, 4, 7, 10 with SRT two to three fractions on week 5–6	Ohio State Comprehensive Cancer Center
NCT02115139	Melanoma (brain metastases)	One arm: ipi followed by WBRT	II	One year survival	Ipi weeks 1, 4, 7, 10 with WBRT between cycles 1 and 2	Grupo Español Multidisciplinar de Melanoma
NCT01860430	Head and neck (stage III–IV)	One arm: IMRT with cetuximab and dose escalating ipi	II	Maximum tolerated dose	IMRT weeks 2–8 (70–74 Gy), Cetuximab weeks 1–8, ipi weeks 1, 5, 8, 11, 14	University of Pittsburgh/NCI
NCT02097732	Melanoma (Brain Metastases)	Two arms: (A) SRT followed by ipi (B) ipi then SRT then ipi	II	Progression-free survival	(A) SRT followed by 4 cycles ipi (B) 2 cycles of ipi then SRT then 2 cycles ipi	University of Michigan Cancer Center

*Ipilumimab(ipi); Radiation Therapy (RT); Sterotactic Radiotherapy (SRT); Stereotactic Radiosurgery (SRS); Whole Brain Radiotherapy (WBRT); Intensity Modulated Radiation Therapy (IMRT); National Cancer Institute (NCI)*.

## Neutralization of the Immunosuppressive Tumor Microenvironment

The immunosuppressive tumor microenvironment is one of the primary means of immune evasion by tumors, yet there are a few ongoing or completed studies combining this treatment approach with RT. The use of low-dose cyclophosphamide to deplete intratumoral Tregs is one example of this approach, and is sometimes used in combination with other immunotherapies. Another interesting study is testing tadalafil with RT. Tadalafil is a small molecule inhibitor of phosphodiesterase 5, which results in inhibition of myeloid-derived suppressor cell function and can target the suppressive myeloid response associated with hypofractionated radiation. An ongoing study for patients with locally advanced and borderline resectable pancreatic cancer is testing the combination of tadalafil with three fractions of 10 Gy delivered every other day to the primary tumor and grossly involved nodes, started after a 21 day cycle of gemcitabine, and patients with resected, stable, or responding disease continue on to receive an additional three cycles of gemcitabine. Like the other early phase studies, the primary endpoints of this study are feasibility and safety, with secondary endpoints looking at immune-correlates from blood and serum samples as well as immunohistology of resected tumor specimens and pathologic response rates (ClinicalTrials.gov identifier: NCT01903083).

## Clinical Approaches: What have We Learned?

Encouraging albeit preliminary results of combining RT and immunotherapy prompt a pause for reflection to take stock of what we have learned so far. Probably, the most promising results are from approaches enhancing the effector function of T-cells primed by RT. Combinations of RT with therapeutic vaccination have shown a more modest promise. The immunosuppressive effect of the tumor microenvironment is one potential reason for this. Vaccination, like RT, can induce priming of tumor reactive CTLs, but given alone it may not be able to overcome local immune suppression in the tumor. Future combinations of cancer vaccines with immunotherapeutics that enhance T-cell function or modulate the tumor microenvironment may prove to be more effective.

One approach that has not been adequately explored is the use of immunotherapeutics to modify the immune-suppressive tumor microenvironment prior to RT. RT has its own local effects on the tumor microenvironment, modifying regulatory lymphocytes, and recruiting new naïve myeloid cells such as DCs and TAMs. In established tumors, MDSCs are another targetable suppressive cell type that inhibit anti-tumor immunity. Modulation of the tumor microenvironment to counteract suppressive elements has the potential to act synergistically with RT to boost the systemic anti-tumor-immune response.

So far, several variables seem to be relevant to the success of combining immunotherapy and RT. Among them, dose and fractionation, site of irradiation, and sequencing with the selected modality deserve further discussion. Dose and fractionation are important factors in the immunogenicity of RT. Pre-clinical data suggests that when combined with CTLA-4 antibody antagonists, 8 Gy in three fractions or 6 Gy in five fractions are superior to standard fractionation or a single dose of 20 Gy (105). The underlying mechanism that explains the difference in immune effect among different dose and fractionation schedules is unclear, but these schedules are supported by the recent clinical reports of impressive abscopal effects after palliative RT to a single metastatic site in malignant melanoma (9.5 Gy × 3) and non-small cell lung cancer (6 Gy × 5) (101, 102).

The target site of RT may be another important consideration when combining RT with immunotherapy. Pre-clinical models are less instructive here, since most models involve radiation to tumors implanted into the subcutaneous tissue. However, when reviewing the clinical reports of abscopal effects, these were observed after irradiation targeting visceral metastases (97–99, 183–188).

The timing of RT relative to immunotherapy is another important consideration. This question has not been addressed thoroughly in the pre-clinical models. In studies combining CTLA-4 blockade with RT using a mouse model of breast cancer, the antibody was administered at different time-points with the best abscopal response seen when the first dose of antibody was given during RT (105). Similarly, the patient with non-small cell lung cancer who experienced an abscopal effect had received concurrent ipilimumab and radiation (102). Yet, the reported abscopal effect in a patient with metastatic melanoma occurred after long-term treatment with ipilimumab prior to RT (101).

Tumor burden and the associated degree of immunosuppression also play an important role in selection of the best candidates for trials combining radiation and immunotherapy. Metastatic tumor burden correlates with immune suppression, probably both as a marker of a weakened immune system and as an active player in systemic immune dysfunction (189, 190). The combination of ipilimumab with RT in men with castration-resistant prostate cancer resulted in a survival benefit only in patients with smaller burdens of metastatic disease, demonstrated by alkaline phosphatase less than 1.5 the upper limit of normal, hemoglobin greater than 11 g/dL, and absence of visceral metastases (182). Perhaps future trials should initially focus on patients with more limited metastatic disease.

Prior conventional therapy may also impact the results of immunotherapy trials. Many chemotherapeutic regimens cause myelosuppression, which depletes the very cells that are necessary for an effective immune response (191). However, some chemotherapeutic agents can cause immunogenic cell death and promote anti-tumor immunity (192). Also, despite the anti-tumor-immune promoting effects of RT, prior irradiation may lead to modification of the tumor microenvironment leading to a more immune-tolerant phenotype (113, 193). The net effect of these prior treatments is not clear, but it is likely to have an impact on the immune system and on the effectiveness of cancer immunotherapy.

Even something as fundamental as defining appropriate clinical endpoints is undergoing a critical re-appraisal, determining the best way to monitor the immune response to these combinations of immunotherapy and RT is an unresolved question. Specific immune responses are notoriously difficult to identify and track since every tumor has a unique complement of mutations and every patient has a unique MHC haplotype for presenting tumor antigens. As a surrogate to immune response and an alternative to the traditional RECIST criteria used to measure the effect of cytotoxic therapy, Wolchok et al. have introduced the immune-related response criteria (194, 195). These criteria take into account the mixed nature of clinical responses to immunotherapy, with some lesions responding while other lesions remain stable or even appear to progress. Importantly, overall survival and toxicity profiles, with their impact on quality of life, have emerged as the main clinical outcomes for immunotherapy. In some trials of immune monotherapy, most notably with sipuleucel-t, no objective response was observed; however, there was a significant improvement in overall survival (196). Multidisciplinary efforts to define optimal immunomonitoring are currently ongoing.

## Conclusion

Ten years ago our group reported the first pre-clinical studies of the systemic anti-tumor effects of RT in combination with modern immunotherapy (104), after providing an immunological explanation for the abscopal effect (104). Now, a decade later, there are over 50 ongoing and published clinical trials combining RT and immunotherapy for the treatment of cancer, with more studies in the pipeline. Future directions may combine multiple approaches to immunotherapy that augment the effect of RT on anti-tumor T-cell priming as well as contribute to other steps of immune rejection (197). Many questions remain with regards to the optimal way to harness ionizing radiation in combination with immunotherapy, and how to best select patients for this approach, determining the most appropriate clinical characteristics, tumor pathology, and stage. Despite all of these challenges, the burgeoning interest in the combination of immunotherapy and RT will provide exciting new insights and avenues to explore as we continue our quest to harness patients’ innate ability to eliminate evasive tumor cells.

## Conflict of Interest Statement

The authors declare that the research was conducted in the absence of any commercial or financial relationships that could be construed as a potential conflict of interest.
